# Generalist Genes: Genetic Links Between Brain, Mind, and Education

**DOI:** 10.1111/j.1751-228X.2007.00002.x

**Published:** 2007-03

**Authors:** Robert Plomin, Yulia Kovas, Claire M A Haworth

**Affiliations:** 1 Social, Genetic and Developmental Psychiatry Centre, Institute of PsychiatryLondon

## Abstract

Genetics contributes importantly to learning abilities and disabilities—not just to reading, the target of most genetic research, but also to mathematics and other academic areas as well. One of the most important recent findings from quantitative genetic research such as twin studies is that the same set of genes is largely responsible for genetic influence across these domains. We call these “generalist genes” to highlight their pervasive influence. In other words, most genes found to be associated with a particular learning ability or disability (such as reading) will also be associated with other learning abilities and disabilities (such as mathematics). Moreover, some generalist genes for learning abilities and disabilities are even more general in their effect, encompassing other cognitive abilities such as memory and spatial ability. When these generalist genes are identified, they will greatly accelerate research on general mechanisms at all levels of analysis from genes to brain to behavior.

Genetic research has moved beyond merely demonstrating the importance of genetic influence. Most notably, intense research efforts are focused on attempts to identify the DNA responsible for this genetic influence, especially for reading disability, and we mention this work briefly. Nonetheless, quantitative genetic research such as twin studies that compare identical and fraternal twins continues to be important in charting the course for molecular genetic explorations. The most important example is multivariate genetic analysis, which makes it possible to investigate genetic links between variables rather than focusing on one variable at a time. The major goal of this article is to provide an overview of multivariate genetic research on learning abilities and disabilities, which consistently points to “generalist genes” that have pervasive effects. We also consider the implications of generalist genes for education and for cognitive neuroscience. In order to focus on this topic of generalist genes, we need to forgo presenting other topics important to the field of mind, brain, and education, such as the developmental interface between genes and environment (Plomin & Kovas, in press-a). However, we begin with a very brief overview of research showing substantial genetic influence on learning abilities and disabilities.

## Genetic influence on learning abilities and disabilities

More than 90% of teachers and parents say that they believe genetics to be at least as important as the environment for learning abilities and disabilities (Plomin & Walker, 2003). In the hope that scientists and policy makers also realize the important contribution that genetics makes to learning abilities and disabilities, we offer only a broad overview concerning this rudimentary nature–nurture issue.

Two decades of research make it clear that genetics is a surprisingly large part of the answer to why children differ in their ability to learn in school. Most research uses the twin method that compares resemblance for genetically identical twins (identical, monozygotic [MZ]) and for twins who are only 50% similar genetically (fraternal, dizygotic [DZ]). Genetic influence is suggested to the extent that MZ twins are more similar than DZ twins, reflecting the twofold greater genetic similarity of MZ as compared with DZ twins. For example, a review of twin studies of language disability reported concordance (the likelihood that one twin will be affected if the other twin is affected) of 75% for MZ twins and 43% for DZ twins (Stromswold, 2001). For reading disability, the average concordances for MZ and DZ twins are 84% and 48%, respectively. For mathematics disability, the concordances are about 70% for MZ twins and 50% for DZ twins (Oliver et al., 2004). Such research suggests not only that genetic influence on learning disabilities is significant but also that it is substantial.

Moreover, genetics is not only involved in disability. Twin studies also consistently point to substantial genetic influence for learning abilities throughout the normal distribution of individual differences. A review of twin studies that reported results for both learning disabilities and abilities found that the average weighted heritability (proportion of phenotypic variance that is attributed to genetic variance) was 0.43 for language disabilities and 0.25 for language abilities; 0.52 and 0.63 for reading disabilities and abilities, respectively; and 0.61 and 0.63 for mathematics disabilities and abilities, respectively (Plomin & Kovas, 2005).

The case for substantial genetic influence on learning disabilities is so clear that most genetic research, especially in the area of reading disabilities, now focuses on using molecular genetics to identify the specific genes responsible for this genetic influence. Although progress has been slow, recent developments in molecular genetics are promising (Grigorenko, 2005; Plomin, 2005;). For reading disability, four candidate genes are currently the targets of intense research (Fisher & Francks, 2006; McGrath, Smith, & Pennington, 2006;). Reports are also beginning to appear of genes associated with normal variation in learning abilities (Plomin, Kennedy, & Craig, 2006). Although this research is exciting, quantitative genetic research, especially multivariate genetic analysis, still has much to offer in guiding molecular genetic research toward the most heritable components and constellations of learning disabilities and abilities.

## Multivariate genetic analysis

Univariate genetic analysis uses methods such as the twin method to estimate genetic and environmental contributions to individual differences (variance) on a single variable (univariate). If MZ twins are more similar than DZ twins for a trait, this suggests that genetic differences account for some of the observed (phenotypic) differences on the trait. Heritability estimates the extent to which genetic variance accounts for phenotypic variance. In contrast, multivariate genetic analysis focuses on the covariance (correlation) between two traits (bivariate) or multiple traits (multivariate) and uses the twin method to estimate genetic and environmental contributions to their covariance as well as the variance of each trait. In other words, multivariate genetic analysis estimates the extent to which genetic and environmental factors that affect one trait also affect another trait.

The gist of multivariate genetic analysis lies in cross-trait twin correlations. Just as univariate genetic analysis compares MZ and DZ correlations for a single trait, multivariate genetic analysis compares MZ and DZ correlations across traits, called cross-trait twin correlations. To the extent that MZ cross-trait correlations are greater than DZ cross-trait correlations, this suggests that genetic differences mediate the phenotypic correlation between the traits.

**Fig. 1:**
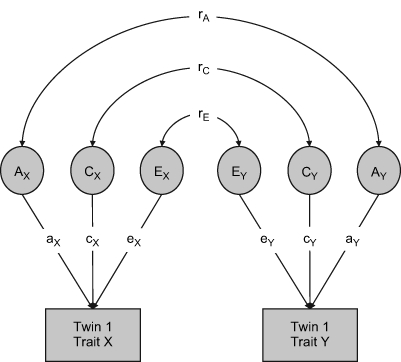
Multivariate genetic model for trait X and trait Y for one individual from a twin pair. (The actual model depends on cross-trait twin correlations for MZ and DZ twins, but the other member of the twin pair is not shown here.) Variance in each trait is divided into that due to latent additive genetic influences (A), shared environmental influences (C), and nonshared environmental influences (E) with the subscripts X and Y denoting scores on traits X and Y, respectively. Paths, represented by lowercase letters (a, c, and e), are standardized regression coefficients and are squared to estimate the proportion of variance accounted for. For example, a_X_^2^ is heritability, the proportion of phenotypic variance explained by genetic variance. Correlations between the latent genetic, shared environmental, and nonshared environmental influences are denoted by *r*_A_, *r*_C_, and *r*_E._

In practice, multivariate genetic analysis is conducted using structural equation model-fitting techniques based on the model shown in Figure 1. The boxes represent the variance of measured traits X and Y and the circles represent latent variables in which the twin method is used to estimate three contributions to phenotypic variance: additive genetic (A), common or shared environment that makes members of a twin pair similar (C), and the rest of the environment (E). These are univariate concepts in that the twin method is used to decompose the variance of trait X and of trait Y into A, C, and E components of variance.

Multivariate genetic analysis focuses on the phenotypic covariance between traits and decomposing the phenotypic covariance into A, C, and E components of covariance. As indicated in the multivariate ACE model shown in Figure 1, the new concept in multivariate genetic analysis is the correlation between the A, C, and E latent variables for trait X and trait Y. We will focus on the genetic correlation (*r*_A_), which represents the extent to which genetic effects on trait X correlate with genetic effects on trait Y independent of the heritability of the two traits. A genetic correlation of 0.0 indicates that completely different genes affect the traits and a genetic correlation of 1.0 signifies that the same genes affect both traits. In other words, the genetic correlation is the probability that a gene found to be associated with one trait will also be associated with the other trait. An important feature of the genetic correlation is that it is independent of the heritability of the traits. That is, the genetic correlation can be high even if the heritabilities of the two traits are low and vice versa. In the interest of streamlining this article, we will not discuss a second multivariate genetic concept, the genetic contribution to the phenotypic correlation, which is represented in the model as the product of the genetic paths connecting trait X and trait Y (i.e., a_X_*r*_A_a_Y_). We will also not discuss the C and E correlations even though they also tell an interesting story (Plomin & Kovas, in press-b).

## Generalist genes for learning abilities and disabilities

The first multivariate genetic analysis in this area using standard measures of reading and mathematics reported a genetic correlation of 0.98 between reading and mathematics (Thompson, Detterman, & Plomin, 1991). In a recent review, genetic correlations varied from 0.67 to 1.0 for reading versus language (five studies), 0.47 to 0.98 for reading versus mathematics (three studies), and 0.59 to 0.98 for language versus mathematics (two studies) (Plomin & Kovas, 2005). The average genetic correlation between domains was about 0.70. However, most of these studies had sample sizes of fewer than 100 pairs of each type of twin, which is a problem because of the statistical power needed to conduct multivariate genetic analyses.

In a large and representative sample of several thousand pairs of twins, we have focused on multivariate genetic analysis of learning abilities and disabilities in the early school years at 7, 9, and 10 years in a study called the Twins Early Development Study (TEDS; Oliver & Plomin, in press). At each age, performance in the basic academic subjects was assessed by teachers based on year-long evaluations using U.K. National Curriculum (NC) criteria. In addition, reading tests were administered at 7 and 10 years and mathematics tests were administered at 10 years. Two verbal and two nonverbal cognitive tests were administered during each year in order to assess general cognitive ability.

**Fig. 2:**
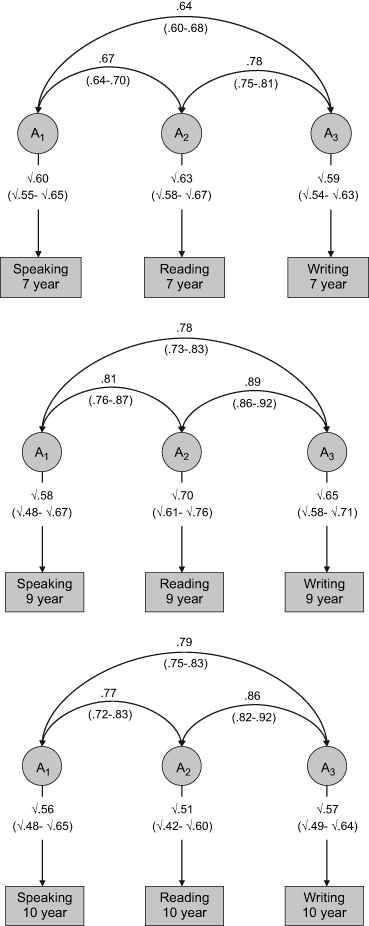
Genetic model-fitting results within three domains of English at 7, 9, and 10 years.

We first investigated genetic correlations for components within each domain at 7, 9, and 10 years. The NC ratings included three domains: English, mathematics, and science. Each NC domain included three components: for example, English included speaking, reading, and writing. Figure 2 presents the multivariate genetic results for English at 7, 9, and 10 years. The genetic correlations between speaking, reading, and writing are 0.70 on average at 7 years and 0.82 on average at 9 and 10 years. Across all three domains across all three ages, the average genetic correlation was 0.78 (Kovas, Haworth, Petrill, & Plomin, in press).

Similar results were found for test scores. At 7 years, the word and nonword components of the Test of Word Reading Efficiency (Torgesen, Wagner, & Rashotte, 1999) yielded a genetic correlation of 0.88. At 10 years, five components of mathematics were assessed including computation, application, and interpretation; these yielded an average genetic correlation of 0.91 (Kovas et al., in press).

**Fig. 3:**
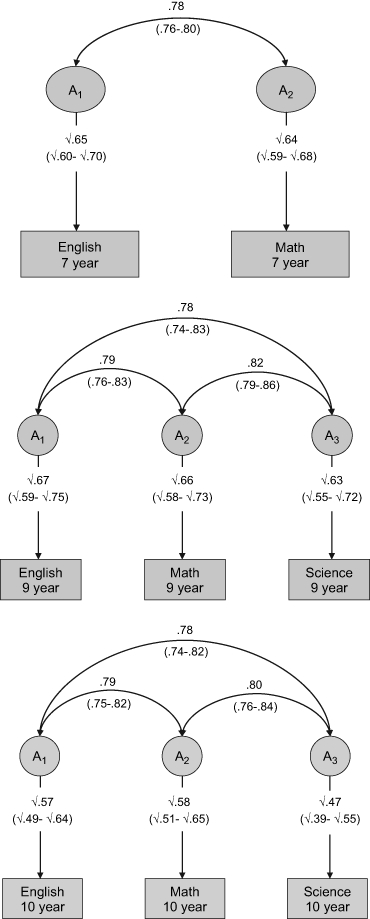
Genetic model-fitting results among English, mathematics, and science at 7, 9, and 10 years.

Even more surprising were the high genetic correlations between domains. In TEDS, we analyzed genetic correlations across NC ratings of English, mathematics, and science at 7, 9, and 10 years (Kovas, Haworth, Dale, & Plomin, in press). Figure 3 summarizes these genetic results. The average genetic correlation was 0.79 among NC ratings of English, mathematics, and science. The genetic correlation was lower but nonetheless significant and substantial (0.52) between the tests of reading and mathematics at 10 years.

These multivariate genetic results suggest that genetic influences within and between academic domains overlap to a great extent. Genetic overlap is surprisingly substantial even between domains such as English and mathematics. In order to highlight these general effects of genes, we refer to them as generalist genes. To be concrete, these results suggest that when DNA research identifies genes responsible for genetic influence on reading ability, most of these genes will also be associated with mathematics ability because the genetic correlation between reading and mathematics is 0.70. Because there is an automatic tendency to square correlations when considering effect sizes, it should be mentioned that the genetic correlation itself, not the squared correlation, indicates genetic overlap between genetic effects on the two traits.

These studies examined the entire distribution of individual differences in learning abilities—what about disabilities? Few multivariate genetic studies of disabilities have been reported because they require large samples of twins for both types of disabilities in order to investigate comorbidity between the disabilities. In general, genetic research comparing abilities and disabilities suggests that what we call learning disability is merely the low end of the same genetic and environmental factors responsible for the normal distribution of learning ability. In other words, the abnormal is normal (Plomin & Kovas, 2005). The implication is that when multivariate genetic studies of disabilities are conducted they will yield similarly high genetic correlations. The first two studies of this sort confirm this expectation. Multivariate genetic analyses of the comorbidity between reading disability and mathematics disability yielded genetic correlations of 0.53 (Knopik, Alarcón, & DeFries, 1997) and 0.67 (Kovas et al., in press), respectively. In summary, these results suggest that generalist genes are relevant to disabilities as well as to the normal distribution of abilities.

A common reaction to this conclusion about generalist genes is disbelief because it goes against the common observation that specific disabilities exist. That is, some children with reading problems have no problem with mathematics and vice versa. If genes are generalists, why do specific disabilities occur? There are three reasons. First, genes are also specialists—genetic correlations are not 1.0. Second, nonshared environments are largely specialists (Plomin & Kovas, in press-b). Third, there is less specificity than it might seem. Even though reading and mathematics correlate phenotypically 0.65 in TEDS, some children with reading problems have no problems with mathematics and vice versa. However, this so-called double dissociation is to be expected on statistical grounds alone and has no bearing on the extent to which different causal processes affect reading and mathematics. A related issue concerning the acceptance of these findings is that, although genetic correlations between learning abilities are greater than their phenotypic correlations, we cannot see genetic correlations in the population in the way that we can see phenotypic associations and dissociations.

## Generalist genes for other cognitive abilities

Much more multivariate genetic research has focused on cognitive abilities such as verbal, spatial, and memory abilities as compared with learning abilities. This research consistently finds genetic correlations greater than 0.50 and often near 1.0 across diverse cognitive abilities (Deary, Spinath, & Bates, 2006). Similar results suggesting substantial genetic overlap have been found for more basic information-processing measures such as speed of processing as well as measures of brain volume (Deary et al., 2006). Genetic overlap across cognitive abilities becomes stronger across the life span (Petrill, 2002).

Phenotypic correlations among diverse tests of cognitive abilities led Charles Spearman in 1904 to call this general factor *g* in order to avoid the many connotations of the word intelligence. To what extent do generalist genes for *g* overlap with generalist genes for learning abilities? A review of about a dozen such studies concluded that genetic correlations between *g* and learning abilities (mostly reading) are substantial but somewhat lower than the genetic correlations among learning abilities (Plomin & Kovas, 2005). For example, in TEDS, an analysis of nearly 3,000 pairs of 7-year-old same-sex twins reported genetic correlations of 0.74 between reading and mathematics, 0.58 between reading and *g*, and 0.67 between mathematics and *g* (Kovas, Harlaar, Petrill, & Plomin, 2005). This result suggests that most (but not all) generalist genes that affect learning abilities are even more general in that they also affect other sorts of cognitive abilities included in the *g* factor.

However, generalist genes for learning abilities and disabilities are not just *g*. This can be seen more directly using a different type of multivariate ACE genetic model, called the Cholesky model, which tests for common and independent genetic and environmental effects on the variance and covariance between traits. The Cholesky procedure is similar to hierarchical multiple regression analyses in nongenetic studies, where the independent contribution of a predictor variable is assessed after accounting for its shared variance with other predictor variables. The Cholesky model is an algebraic transformation of the correlated factors model shown in Figures 1–3 and yields the same results such as genetic correlations.

**Fig. 4:**
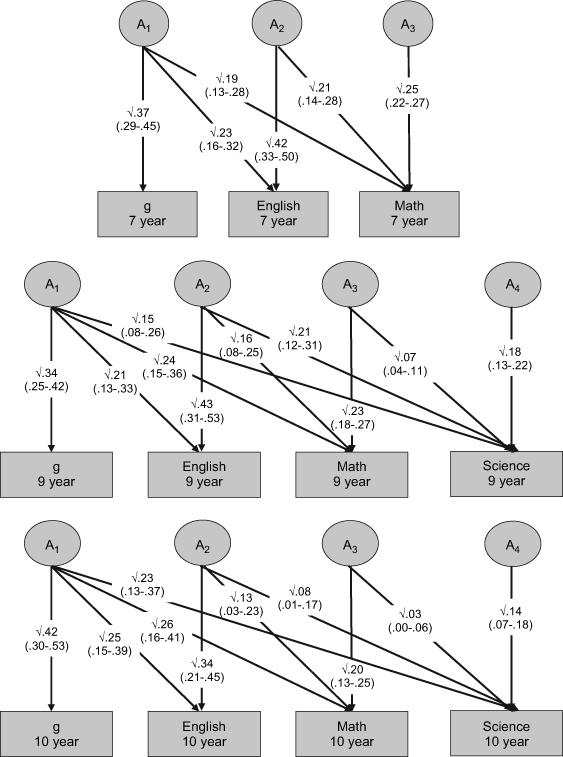
General cognitive ability (*g*) and learning abilities: genetic model-fitting results among *g*, English, mathematics, and science at 7, 9, and 10 years.

Using TEDS data on *g* (a composite of two verbal and two nonverbal tests) and NC ratings for English, mathematics, and science, we conducted separate Cholesky analyses at 7, 9, and 10 years. The genetic results are presented in Figure 4. The results at 7 years are presented in the first panel of Figure 4. The A_1_ latent variable extracts genetic variance that is in common between *g* and academic performance in English and mathematics. The heritability of *g* at 7 years is shown as 0.37. The A_1_ loadings of 0.23 for English and 0.19 for mathematics indicate that a significant and substantial amount of the genetic variance on English and mathematics is shared in common with *g*. However, English and mathematics are more highly heritable than *g*: The heritability estimates from Figure 4 are 0.65 for English (i.e., 0.23 + 0.42 = 0.65) and 0.65 for mathematics (0.19 + 0.21 + 0.25 = 0.65). Thus, only a third of the genetic variance on English is shared in common with *g* (0.23/0.65 = 0.35). Similarly, only a third of the genetic variance on mathematics is shared in common with *g* (0.19/.065 = 0.29).

An important feature of the Cholesky model is that it estimates genetic variance shared by English and mathematics that is independent of *g*. This analysis is captured by the A_2_ latent variable. The significant and substantial loadings of English and mathematics on the A_2_ latent variable indicate that English and mathematics share genetic variance independent of *g*. For mathematics, about a third of its genetic variance is shared with English independent of *g* (0.21/0.65 = 0.32). The A_3_ latent variable indexes genetic variance that is unique to mathematics, that is, not shared with either *g* or English. Focusing on mathematics, the results suggest that about a third of its genetic variance is in common with both *g* and English, about a third is in common with English independent of *g*, and the remaining third is unique to mathematics. A similar conclusion would be reached for English if it were the last variable in the Cholesky analysis.

Similar results were obtained at 9 and 10 years, as shown in the second and third panels of Figure 4, when NC ratings for science were also available. Focusing on science, which is the last variable in the Cholesky analysis, the heritability of science at 9 years is estimated in this model as 0.61 (0.15 + 0.21 + 0.07 + 0.18 = 0.61). Of the genetic variance on science, 25% is in common with *g*, English, and mathematics (0.15/0.61 = 0.25); 34% is independent of *g* but in common with English and mathematics (0.21/0.61 = 0.34); 11% is independent of *g* and English but in common with mathematics; and 30% is unique to science.

At 10 years (third panel of Figure 4), the heritability of science is estimated as 0.48, lower than the estimate of 0.61 at 9 years. Of this genetic variance, 48% is in common with *g*, English, and mathematics; 17% is independent of *g* but in common with English and mathematics; 6% is independent of *g* and English but in common with mathematics; and 29% is unique to science. This suggests that science at 10 years may have more to do with *g* genetically. However, the results for English and mathematics are similar at 10 years in suggesting that only about a third of their genetic variance is shared in common with *g*.

The main point of these analyses is that academic performance is not just *g*. That is, although about a third of the genetic variance of English and mathematics is in common with *g*, about a third of the genetic variance is general to academic performance but not *g* and about a third is specific to each domain.

## Implications of generalist genes

The implication of generalist genes for molecular genetic research is clear: Most (but not all) genes found to be associated with a particular learning ability or disability (such as reading) will also be associated with other learning abilities and disabilities (such as mathematics). In addition, most (but not all) of these generalist genes for learning abilities (such as reading and mathematics) will also be associated with other cognitive abilities (such as memory and spatial).

### Implications for education

Although finding generalist genes associated with learning disabilities is unlikely to have much direct impact on teachers in the classroom confronted with a particular child with a learning disability, such findings will have far-reaching ramifications in terms of educational research and, more practically, in terms of diagnosis, treatment, and prevention. At the most general level, identifying generalist and specialist genes will increase acceptance of genetic influence in education because DNA provides evidence for genetic influence that is much more direct than the evidence provided by quantitative genetic research such as twin studies.

In terms of research, few educational researchers are likely to become involved in the quest to find genes associated with learning abilities and disabilities, but when the genes are found, they will be widely used in research as DNA risk indicators in much the same way that demographic risk indicators are currently used (Plomin & Walker, 2003). Moreover, DNA has a unique causal status in that correlations between DNA differences and behavioral differences can only be explained causally in one direction: DNA differences cause behavioral differences. This causal status of DNA is unique in the sense that correlations involving other biological variables such as brain variables are just correlations that can be explained in either causal direction—behavioral differences can cause brain differences. However, variation in DNA sequence, which is the basis of heredity, is not changed by behavior, biology, or the environment.

The most immediate implication for education is the realization that genetic diagnoses of learning disabilities differ from traditional diagnoses, which are based on symptoms rather than causes. From a genetic perspective, learning disabilities are not distinct diagnostic entities: the same set of generalist genes affects learning abilities and disabilities. Finding generalist genes associated with learning disabilities will lead to new diagnostic classifications that are based on etiology rather than symptomatology.

In terms of treatment, genes will be used clinically or educationally to the extent that response to treatment depends on genetic risk. This goal is part of a “personalized medicine” movement toward individually tailored treatments rather than treatments that are “one size fits all” (Abrahams, Ginsburg, & Silver, 2005).

The most important benefit of identifying genes that put children at risk for developing learning disabilities is that the causal nature of genes means that they can serve as an early-warning system. This should facilitate research on interventions that prevent learning disabilities, rather than waiting until problems are so severe that they can no longer be ignored. The goal of early intervention fits with a general trend toward preventative medicine. Because vulnerability to learning disabilities involves many genes of small effect, genetic engineering is unimaginable for learning disabilities; interventions will rely on environmental engineering, primarily educational interventions.

### Implications for brain and mind

Acceptance of generalist genes will change the way we think about the brain and mind. In this final section, we discuss two genetic concepts—pleiotropy and polygenicity—that provide a foundation for understanding the effects of generalist genes on the brain and mind.

Pleiotropy means that a gene has multiple effects. In terms of individual differences—which is the focus of genetic studies on learning abilities and disabilities—polymorphisms in these genes will also have pleiotropic effects. Pleiotropy is common in complex organisms and can be expressed at various biological levels, from a gene that mediates several intracellular signal transduction pathways to a gene that is expressed in different tissues (Dudley, Janse, Tanay, Shamir, & Church, 2005). As one of hundreds of examples, most of the genes responsible for the 185 proteins involved in the NMDA (N-methyl-D-aspartic acid) receptor complexes are widely expressed throughout the central nervous system (Grant, Marshall, Page, Cumiskey, & Armstrong, 2005).

A powerful new tool for seeing pleiotropy in the brain is gene expression mapping. Gene expression can be indexed by the presence of RNA that is transcribed from DNA. A critical development in gene-expression mapping throughout the brain is the microarray that can detect the expression of all the genes in the genome simultaneously (Greenberg, 2001), which is the genetic equivalent of neuroimaging. In the present context, the key question is the relative specificity or generality of gene expression across brain regions at the level of individual differences, not at the normative level, which is the level of analysis that pervades such research. That is, when allelic variation in a gene is found to be associated with individual differences in reading ability, will this allelic variation be associated with just one brain region or with many brain regions? The generalist genes hypothesis predicts that the effects of individual differences in gene expression are distributed widely throughout the brain rather than being localized in a specific region.

Although in its early stages with scarcely any research at the individual–differences level of analysis, gene-expression mapping so far supports the generalist genes hypothesis in that most genes are expressed throughout the brain, not just in one specific region. For example, two genes most often studied in human cognition are catechol-*O*-methyltransferase (COMT) and brain-derived neurotrophic factor (BDNF) (Plomin et al., in press). COMT is one of the major metabolic pathways of the catecholamine transmitters; BDNF is a member of the nerve growth family and is induced by cortical neurons. Because both genes have such basic neural functions, it seems likely that their effects in the brain are widespread both in terms of structure and function. This expectation is supported by gene-expression brain maps: In humans as well as mice, both COMT and BDNF are expressed in cortex, cerebellum, caudate nucleus, amygdala, thalamus, corpus callosum, dorsal root ganglia, and spinal cord (see www.geneatlas.org; www.brainatlas.org) (Su et al., 2004).

Similar to early neuroimaging research, genetic neuroimaging work has focused on structural localization at the normative level of analysis, for example, in the transcriptome-mapping project in humans (Yamasaki et al., 2005). Structural brain maps of gene expression are fundamental because genes can only function if they are expressed (i.e., transcribed from DNA to RNA). The next step will involve the much more difficult task of functional genetic neuroimaging—studying changes in gene expression as a function of interventions such as learning and memory tasks or drugs. One problem is that, in the human species, localization of gene expression can only be studied using postmortem brain tissue, which limits research to structural genetic neuroimaging. Functional genetic neuroimaging needs to rely on mice and other animal models. Mouse research is obviously not useful as a behavioral model of uniquely human behaviors such as reading and mathematics. However, mouse model research will play an important role in charting the functional expression of genes in the brain, including genes for learning abilities and disabilities, because nearly every human gene can be found in only slightly altered form in mice. Research on mice is underway that aims to create an atlas of patterns of gene expression throughout the brain during learning and memory tasks (Grant, 2003) in the Genes to Cognition research consortium (see www.genes2cognition.org). The generalist genes hypothesis predicts that functional genetic neuroimaging will also show general effects of allelic variation across brain regions and across tasks.

The effects of pleiotropy (each gene affects multiple traits) are amplified by polygenicity (each trait is affected by multiple genes). Again, much of the discussion of pleiotropy is at a normal level rather than the individual-differences level, which is the critical level of analysis for learning abilities and disabilities. For common disorders and complex traits, genetic research on individual differences has undergone a revolution that has radically altered molecular genetic strategies. Instead of thinking about rare genetic disorders caused by a single-gene mutation of the sort that Mendel investigated in the pea plant, it is now generally accepted that common disorders are caused by many genes (polygenicity), which implies that each of these genes will have only a small effect. Single-gene disorders are usually extremely rare, with a frequency of one in tens of thousands, whereas the frequency of learning disabilities such as reading and mathematics disability is often considered to be as great as 5%. It is likely that a few cases of learning disability are due to single-gene disorders that contribute little to normal variation in learning ability. However, most researchers now believe that common disorders are caused by common genetic variants—the common disorder/common variant hypothesis (Collins, Euyer, & Chakravarti, 1997)—rather than by a concatenation of rare single-gene disorders.

Polygenicity is thought to be the reason why progress has been so slow in identifying genes associated with common disabilities and complex traits—very large samples are needed to attain the statistical power needed to detect very small effect sizes (Plomin, 2005). In the present context, the point of polygenicity is that when we refer to generalist genes we do not mean one or two or even a few genes but perhaps hundreds of genes, each with tiny effects on average in the population.

In our opinion, pleiotropy and polygenicity make it likely that generalist genes result in “generalist brains” at the individual differences level of analysis. That is, polymorphisms in genetic input into brain structure and function have general effects, not modular effects (Kovas & Plomin, 2006). In other words, if multivariate genetic analyses of brain structure and function were conducted similar to those presented in this article at the behavioral level, we predict that genetic correlations would be substantial. However, identifying specific genes associated with learning abilities and disabilities will provide definitive proof that the effects of generalist genes on learning abilities and disabilities are mediated by generalist brains.

Pleiotropy and polygenicity will make it difficult to investigate links between genes, brain, and behavior. Despite these challenges and complexities, when generalist genes are identified, they will provide three opportunities for empirical research on brain pathways between genes and learning abilities and disabilities. First, these genes will be identified on the basis of their prediction of learning disability. In other words, no matter how complex the brain pathways are between genes and learning abilities and disabilities, these genes will be anchored to a functional effect at the level of individual differences in learning abilities and disabilities. Second, each of these generalist genes will provide a window through which we can view brain mechanisms that are functionally related to learning disabilities (Fisher, 2006). Third, although there are likely to be many genes of small effect size, rather than fractionating the view of brain processes, these generalist genes will provide glimpses of processes that are all united functionally in terms of their ultimate effect on individual differences in learning abilities and disabilities. We hope that research on generalist genes as well as specialist genes will lead to greater understanding of the genetic links between mind, brain, and education.
